# Unusually Located Stroke After Chemotherapy in Testicular Germ Cell Tumors

**DOI:** 10.1177/2324709615590198

**Published:** 2015-06-09

**Authors:** Braulio Alexander Martinez, Edgar Patricio Correa

**Affiliations:** 1 Department of Neurology, Carlos Andrade Marin Hospital, San Francisco of Quito University, Quito, Ecuador

**Keywords:** testicular cancer, ischemic stroke, chemotherapy, cisplatin

## Abstract

Testicular cancer is a type of malignancy that affects young adults and has high rates of cure; however, as any malignancy, it is associated with an increased risk of ischemic or hemorrhagic cerebrovascular disease, given the systemic tumor effects or side effects of chemotherapy, which in turn increases morbidity, functional impairment, and additional risk of early death.

## Introduction

Patients with systemic cancer have a high risk of suffering arterial or venous thromboembolic events (TEEs), which include deep-vein thrombosis, pulmonary embolus, stroke, and unstable angina/myocardial infarction (MI). The arterial TEEs are less frequent. Studies have shown that the annual incidence of venous thromboembolism in the general population is 117 per 100 000 males per year; however, in cancer patients, it is increased to 1 event in 200 males per year.^1,2^

Up to 90% of cancer patients suffer hemostatic alterations, represented by an increased platelet aggregation, increased activity of procoagulant factors, decreased activity of anticoagulant factors, and increased levels of fibrinogen, which induce a state of hypercoagulability,^3-6^ increasing the risk of ischemic stroke (IS). Nonetheless, there are other influencing mechanisms, such as tumor vascular compression or infiltration and toxic side effects of radiotherapy or chemotherapy (CHT).^6^ A cohort study demonstrated that cancer itself is associated with a higher risk of TEEs (relative risk = 4.1); this risk rises 6.5 times when CHT is added.^7^

The association between anticancer CHT and thromboembolic phenomena was first reported among patients with breast cancer. Since then, similar events have been reported with a variety of anticancer agents.^5^ The associated drugs include cyclophosphamide, methotrexate, 5-fluoracil, and tamoxifen. Association with cisplatin and bleomicin has also been described. These last 2 drugs produce vascular toxicity as a collateral effect.^3,5,6,8,9^ The TEEs can result in higher morbidity rates, impaired quality of life, and, in some cases, may put the patient’s life at risk.^5^

Germ cell tumors (GCT) are relatively infrequent. They represent 1% of malignancies in the United States. CHT cures 90% of the patients and increases the survival rate in up to 80% of the cases, but this can be affected by vascular complications.^5,8,10-12^

Testicular GCTs are the most common solid tumors in men between ages 15 to 35.^11-14^ Randomized studies have shown that PEB-based CHT (cisplatin, etoposide, and bleomicin) is the key to the treatment. However, cisplatin is related to arterial TEEs,^8,10,11,13,15-18^ which include thrombotic microangiopathy, MI, and stroke.^19-22^ This risk is increased in patients who have hepatic metastasis or who receive high doses of steroids.^23^

Many reports have been written about ischemic or hemorrhagic stroke that occurred several days after cisplatin infusion and which have possible etiological factors related with vascular injury, altered platelet aggregation, or increased plasma levels of von Willebrand factor antigen (vWF:Ag).^3,6,22,24^

We report the case of a patient with a testicular seminomatous tumor, who received PEB-based CHT and developed an IS located in the diencephalon and mesencephalon, the present case being different to other published cases in which great vessel infarctions affecting the brain hemispheres are reported.

## Clinical Case

The patient was a 28-year-old man, with a 1-year diagnosis of a right-sided testicular tumor, without cardiovascular risk factors or any other medical condition. In his family history he had a brother who died from testicular cancer at the age of 38 years. The patient underwent a right orchiectomy, and a histopathological study reported a classic seminoma. Preoperative tumor markers such as HCG (human chorionic gonadotropin) and lactic dehydrogenase (LDH) were elevated, while α-fetoprotein levels were normal. Computed tomography of the abdomen reported retroperitoneal lymphatic nodes. Therefore, it was classified as a classic stage IIB seminoma with a good prognosis following the criteria of the International Germ Cell Cancer Collaborative Group. The cardiopulmonary parameters of the patient, which included an electrocardiogram and pulmonary function, were normal. Two months after the orchiectomy, treatment with standard PEB-based CHT was established. After the first cycle, the patient presented febrile neutropenia, which he overcame. There were no complications during the second cycle. Twenty-four hours after the third cycle of CHT, he presented nausea and vomiting. At 48 hours, he had diplopia, and at 72 hours he presented alteration in the level of awareness. Initially, the patient exhibited somnolence that escalated to stupor after 12 hours. In the neurological exam a palsy of the third right nerve was found. Furthermore, he presented tetraparesia mostly in his right limbs and bilateral Babinski reflex.

**Figure 1.**
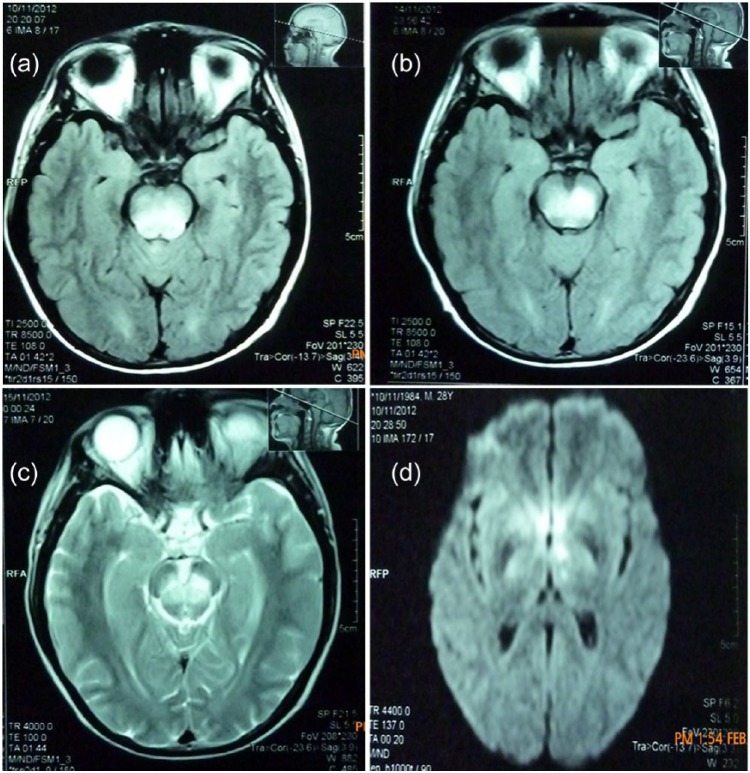
Acute IS within the pons on T1-weighted imaging (a), midbrain on T1- and T2-weighted imaging (b and c), and left thalamus on diffusion (d).

The patient was hospitalized under the suspicion of brain metastasis. For this reason, a magnetic resonance imaging simple with contrast agents was prescribed, which demonstrated the presence of a T1 and T2 hyperintense signal with restricted diffusion in the posterior portion of the pons, the midsection of the mesencephalon, and the ventromedial region of the thalamus (Figure 1), which is compatible with infarction. In addition, metastasis to the brain were not found.

The patient had normal blood pressure and heart rate values. On day 15 of hospitalization, the patient presented a dystonic position of the right arm, and a mixed postural-kinetic tremor. In order to determine the cause, the following laboratory tests were prescribed: clotting times, fibrinogen, plasminogen, α-2-antiplasmin, antithrombin III, protein C, protein S, magnesium, renin, and complement, which were all within normal range. Additionally, antiphospholipid antibodies, antinuclear antibodies, venereal disease research laboratory, human immunodeficiency virus, and hepatitis B and C tests were negative. Because of the lack of resources in our hospital, the vWF:Ag level could not be determined.

Magnetic resonance imaging spectroscopies showed an injury with high levels of lipids and lactate and lowered levels of aspartate, findings that are associated with an infarction area. Magnetic resonance angiography and catheter angiography did not show any injury in large arteries or cerebral vasculitis. The transesophageal echocardiography was normal, and the Holter monitoring did not reveal paroxysmal atrial fibrillation.

After approximately 6 months, the patient was admitted into the Internal Medicine department due to a septic shock secondary to pneumonia and infected bed sores, and due to these infections, he died.

## Discussion

The incidence of testicular cancer varies considerably around the world. In the United States, nearly 7200 cases are diagnosed every year, and the incidence adjusted by age is 5.2 per 100 000 males per year; on the contrary, in European countries the incidence has increased from 2% to 5% per year. The risk is 4 times greater in white individuals than in black individuals.^25^ Ninety-five percent of all malignant testicular tumors are GCTs, and they are classified into 2 major types, seminomatous (40%) and nonseminomatous (60%); the advances in treatment in the past few years have improved disease-free survival rates, with a low mortality rate of 0.38 per 100000 cases per year. However, survivors have been affected by the secondary effects of the CHT used, such as late toxicity, which has led this group of patients to develop a significant associated morbidity rate.^10,11,14,15,18,25^ The cause of GCT is unknown. However, familial clustering has been observed, and factors like cryptorchidism and Klinefelter’s syndrome have been mentioned.^14,18,25^ In the case of our patient, there is a family history (brother).

When testicular cancer is suspected, inguinal orchiectomy is the standard treatment. Independent of the tumor stage, seminomas have high rates of cure, from 100% for tumors in stage I to 95% for stages II and III.^18,26^ In stage IIB (our patient) and after the orchiectomy, CHT is the standard treatment. When CHT is administered, it is based on cisplatin, which is the cornerstone of the standard treatment, and it consists of 3 cycles of administration of the PEB scheme.^10,11,14,15,17,18^

Even though cisplatin is part of the treatment of seminomas, this drug appears as the major cause of long-term toxicity (late) in patients treated for testicular cancer, producing nephrotoxicity, ototoxicity, neurotoxicity, and vascular toxicity, among others.^13,17,21,27^ IS has also been identified as a complication, although its appearance, as in the present case, is rare.^24^ So a retrospective study that included 932 patients with cancer of different etiology (39 patients with GCT) treated with cisplatin-based CHT found that the incidence of arterial or venous TEEs between the first dosage and 4 weeks after the last dosage was 18.1% (169 patients), of which 1.1% (10 patients) were IS events.^22^

There are few reports written about cerebrovascular events related to the administration time of CHT, as in this case in which the patient developed an IS 48 hours after the third PEB cycle. This finding is similar to the case reported by El Amrani et al, where 5 patients with oropharyngeal cancer presented IS within 2 to 5 days after receiving cisplatin and 5-flouracil.^28^ In reported cases of GCT, this time relationship has also been observed, such as the one presented by Vos et al, in which a 30-year-old patient suffered 3 occlusive-arterial infarcts after 10 days of each CHT cycle.^26^ Another case report by Karagoz et al presented a 34-year-old patient who developed aphasia and hemiplegia 10 days after the third CHT cycle,^29^ and the report of Santos et al, whose 20-year-old patient developed hemiparesis and hemihypoesthesia 14 days after the second CHT cycle.^3^ Azak et al described the case of a 17-year-old patient who presented an IS 7 days after the fourth CHT cycle. With this case, in particular, the ischemic events were hemorrhagic infarctions.^30^ Gerl et al described a case of a fatal IS post-CHT, but we could not get major details from that case.^31^ As can be appreciated, the majority of the ischemic events occurred within 10 days after receiving a CHT cycle. In every case described above, there were no atherosclerotic cardiovascular risk factors, as demonstrated by Doll et al in 4 patients under 30 years of age with GCT who were receiving CHT and suffered ischemic vascular events (2 MIs and 2 ISs), in which no cardiovascular risk factor was found.^32^ It calls for attention that our case, as with the other IS events previously described, occurred in young patients, contrasting with the literature description in which the majority of ischemic events in patients in CHT occur in the fifth and sixth decade of life.^29^

The pathophysiological mechanism of cerebral infarction in these cases is not known, but many theories suggest that it is a direct effect of CHT. It has been proposed that cisplatin is associated with vascular injury, platelet-aggregation alterations, augmented serum levels of vWF:Ag, and autonomic dysfunction. The last factor would lead to an augmented α-adrenergic tone that, combined with a state of hypomagnesemia, could cause a vascular spasm.^3,19,21,24,33^ Bleomicin, on the other hand, would cause endothelial alterations in capillaries and arterioles, such as vacuolization, necrosis, and occlusion. Furthermore, the tumor itself can cause thrombosis directly (synthesizing procoagulant factors, factors that modify fibrinolysis, and proinflammatory cytokines) or indirectly (by interaction with platelets, endothelium, and reticuloendothelial cells).^21,34,35^ Finally, it has been proposed that cancer produces embolization of tumor particles, nonbacterial thrombotic endocarditis, and intraarterial precipitation of cryoglobulins.^3,7^

In our case, we performed many studies but we could not establish the etiology of this IS, so it was considered an IS secondary to the received CHT, and as described above, it is possible that bleomicin and cisplatin have influenced its development.

In terms of the localization of the IS caused by CHT, according to reports on cancer patients and specifically in GCT, the condition occurred in larger vessels like the carotid, anterior cerebral, and medial cerebral arteries.^3,29,36-38^ However, in our case the lesion occurred in small-caliber arteries irrigating pons, midbrain, and thalamus, which are anatomic regions and vascular territories in which this entity has never been described, which makes this case unique.

The healing prognosis in this group of patients is high; the affected age-group is young and generally without comorbidity. Furthermore, they were economically active, and for this reason it is important to consider these cerebrovascular complications (whether they are cancer-secondary or above all, secondary to the CHT), because they have the capacity of impairing the patient’s functionality, diminish their quality of life, and, worst of all, lead them to death.

In the future, closer controls should be performed in patients receiving the mentioned CHT schemes and others that have potential vascular toxicity through markers such as the vWF:Ag, which allow us to establish the risk and possibly foresee these complications.^3,33,35^
